# Indents

**Published:** 1912-12-15

**Authors:** 


### INDENTS.

☞Brief Articles of Technical or Scientific Value will receive notice and comment. Contributions invited.

*The Miller Prize Awarded.* At the meeting of the International Dental Federation in Stockholm last August, the Miller medal for eminent service to the dental profession was awarded to Dr. Charles Gordon of Paris, France. Dr. Gordon is not a great scientific worker but he has done much for the advancement of his profession both in France and Internationally.

*Dental Reserve Corps.* The new law creating a staff of commissioned dental surgeons for the Navy carries with it provision for a Reserve Corps of dental surgeons, who shall act only in an advisory capacity and voluntarily when there is a special need. This body will serve without pay and is appointed by the President.

*California in 1915.* A committee from the California Dental Society and the San Francisco Panama Exhibition invited the National Dental Association to meet in San Francisco in 1915 in connection with the Panama Pacific Dental Congress. The invitation was extended at an entertainment given to the members of the Association in the New Willard Hotel, at Washington, and the promises made of unusual facilities and attractions were so convincing that a unanimous decision to accept was carried. There will undoubtedly be a large gathering of dentists on the Pacific Coast that year, as every inducement possible to get them there will be made.

*Medicinal Value of Fruit.* H. M. Lome (*Life and Health*, September, 1912) says that fruit acids are germicidal. The harboring place for many of the most common and dangerous microbes that afflict humanity, is the intestinal tract. The use of citrous fruits is somewhat of a protection against maladies that these microbes cause. As a mouth wash, lemon juice has some virtue. A very dilute solution of the acid can be used with advantage for tired eyes and inflamed eyelids. Scorbutic affections yield to its use. Lemonade is too well known as a refreshing drink to need mention. And as a drink for feverish invalids, it is unsurpassed. It is also good for diabetic patients. Travelers escape tropical fevers by the liberal use of drinks of which lemon or lime-juice is the basis.

Apples, pears and quinces are all members of a botanical family that includes the roses, and is scientifically known as Pyrus malus. Ripe apples eaten raw and thoroughly masticated, are sometimes excellent for digestive troubles. In Devonshire, England, there is an apple-cure establishment for dyspeptics that is said to have effected some remarkable recoveries by placing the patients on an exclusive diet of the fruit. Skin and allied diseases yield to a treatment that includes apples as one of the chief articles of diet. Together with the pear, the apple is a mild aperient. Fresh apple-juice, taken before breakfast, is excellent for constipation. The quince is used only in the form of preserves. Owing to its aggressive astringency when raw, it is sometimes employed to stop hemorrhage by placing slices of it on the wounds.

Unfermented grape juice acts as a mild laxative and diuretic, and diminishes the acidity of the urine. It is, therefore, good for gout, rheumatism, obesity, scorbutic afflictions, kidney troubles, and digestive disorders, including those that have their origin in the liver. And according to Robert Hutchinson, M.D., the famous English doctor, grapes are of the utmost value in the case of chronic bronchial catarrh.

At the European grape-cures, patients consume from one to eight pounds of the fruit daily. The grapes are not used as exclusive diet, but are eaten between meals. Each patient has to gather his own grapes. Doubtless this enforced exercise in the open aids the action of the grapes An American physician who visited one of the French cures, noted that many of the patients were suffering from fatness of the lower part of the body, due to their indulgence in the good things of the table and the habits of inaction. To such persons, the effort of gathering the grapes was an affliction yet a blessing in disguise. It is said that two or three weeks of grape-eating betters the condition of the most of the patients.

Rhubarb, owing to the large proportion of oxalic axid that it contains, is a capital antiscorbutic. In minor forms of scurvy, it acts as a curative. The young plant when stewed and eaten at breakfast is laxative.

Bananas contain more starch than any other known fruit. For this reason, while they are very nutritious, they are not laxative. They may be used with advantage by those who suffer with looseness of the bowels.

The fig is rich in cellulose. On account of this quality it possesses laxative powers of a high order. Confirmed cases of constipation can be cured by the use of sound, dried figs. Many figs offered to the public are moldy, partly rotten, or maggot-eaten, and unfit for consumption. They should be plump, free from a suggestion of mould or blight, and of a fragrant odor.

Peaches, apricots, nectarines, and all the stone-fruits, contain much cellulose, and usually have marked laxative effects. When fully ripe, they have a tonic quality—that "picks up" those of delicate appetite. It is said by some investigators that this bracing effect is due to an infinitesimal quantity of prussic acid, which gives the flavor to the kernel of the fuit and escapes into the pulp. There are many poisons of the deadliest descriptions that, used in microscopic quantities, are of therapeutic value, and it would seem that that of the stone-fruits is one of them.

The plums have medicinal qualities akin to those of the fruits just named. The prune is especially well provided with cellulose, and hence its well-known effects on the organs of excretion.

Cranberries and gooseberries are plentifully supplied with acid, and are of value to those suffering from harsh, rough skin, or from scorbutic affections of any kind. Currants are also endowed with a liberal quantity of acid, but in addition have a very large percentage of fruits-sugar. Therefore they are fitted for diabetic patients as well as for anemic; for in both, such sugar can be used when other kinds of sugar would be harmful.

Iron salts enter largely into the composition of the strawberry, and make that fruit particularly acceptable to those who are nervous and run down. The acid of this fruit is said to be of benefit to sufferers from kidney and bladder troubles. Because of the absence of cane sugar in the strawberry, it also can be safely used by the diabetic.

The pineapple contains a substance that assists in the digestion of food. The pineapple is not suited to diabetics, owing to its containing cane-sugar. But in the case of others, it is of value for its digestive and antiscorbutic properties, and for its stimulative action on the bladder. Also if eaten in liberal quantities on an otherwise empty stomach, it will overcome ordinary constipation.

Dates are mildly stimulating. Tamarinds are markedly laxative. In the British army in the tropics, this fruit, prepared, is served daily for the purpose of insuring regular excretory action. Melons and pumpkins contain a comparatively large proportion of phosphoric acid.

Blackberries, raspberries, huckleberries, and other similar kinds, are rich in acids and cellulose, and act as blood purifiers and laxatives. The cellulose takes the form of the puthy grains that are imbedded in the pulp. These grains cannot be digested. When one eats the fruits, the intestines make a special effort to rid themselves of them; hence the laxative action that usually accompanies the use of berries.

*Lanolin for the Hands.*

Lanolin is an excellent emollient for the hands. Its occasional use will keep the skin smooth and supple; for this purpose it is better than glycerine. Glycerin tends to draw the moisture from the skin, and at times it is irritating, while lanolin is soothing and healing. Scented with a delicate perfume and well rubbed into the skin, after washing the hands, it will keep them in good order. It is not at all expensive, and a little goes a long way. It is especially useful when the hands become chapped, and as a soothing application to the skin cracks so annoying to some during frosty weather.—*John H. Grumm*, Richmond, Va.

*Fighting Tuberculosis by Open Air School Rooms.* Dr. Henry D. Chadwick (*Journal of the Outdoor Life*) calls attention to the important fact that Von Pirquet and other careful investigators have proven that nearly all children under fourteen years of age react to the tuberculin test, showing thereby that they have in some way become infected with tuberculosis. Tuberculous infection in childhood seems to be an automatic process on account of the almost universal distribution of the tubercle bacillus in the homes, schools, and places of amusement, which make up the environment of developing children.

Until a few years ago it was taught in most books of medicine that pulmonary tuberculosis was rare in children under ten or even fifteen years of age. Recent pathological investigation, however, reveals the fact that involvement of the lungs and bronchial glands is the most frequent of all forms of tuberculosis, in children as well as in adults. A tuberculin reaction in older children has a serious significance only when symptoms showing a localization of the disease can be discovered, or in the absence of such signs, if a child has a little rise of temperature in the afternoon, is undeveloped, or anaemic. Such children should be sent to a sanitorium, where they will usually make rapid improvement, and their resistance will be raised to such a degree that serious disease is prevented.

It has already been shown that open-air and open-window schools have been successful in restoring debilitated children to health. It is proposed in many places to have one such room in all modern school houses, and to place therein selected children whose physical condition is impaired. Why not go a step further and do away with the expensive methods of ventilation now in vogue? These systems supply a vitiated atmosphere, superheated and excessively dry, or artificially humidified, which for efficient service demands tightly sealed rooms. In other words, a synthetic preparation containing oxygen is substituted for fresh air as it is freely supplied by nature. Instead of one healthful room in each building to care for such children as are made ill in other rooms (which are now so arranged that they harbor disease and spread infection) why not have all the school rooms of all the buildings built so that air as it comes from nature's laboratory may have free egress and exit?

The present idea of one open-air room in a building bears the same relation to anti-tuberculosis work at the present day as did the attempt in 1898 to cure a few patients who had tuberculosis in an early stage, without making any attempt to isolate the advanced cases, which were allowed to spread infection promiscuously among their friends and associates.

We should give the children who are well a chance to keep well, and in the same way and at the same time, raise the resistance of those who are infected, but not necessarily diseased.

---

Chadwick's idea of a modern school building in which these conditions will be met, is one in which every room is open on at least two sides. In large city schools this could be arranged by a building in the form of a hollow square with a large court yard or play ground in the center. The upper sash of each window should have muslin instead of glass, so that storms and winds could be excluded, but the full passage of air would not be much impeded. Heat could be produced by sufficient radiation under the windows to keep the temperature of the room about sixty degrees. The humidity would approximate that of the outside air.

Under such conditions the teacher and children could keep comfortable without being burdened with heavy clothing. Although fresh air in its natural unchanged condition is what children thrive on, it does not follow that schools need to be conducted out of doors in winter. Cool air is refreshing; cold air is stimulating; but extreme cold is debilitating and dulls the children's mental perceptions. I believe that the degree of cold should be graduated or modified as can be readily done in open-window schools by the method previously referred to.

I hope eventually a hospital school-building will be erected at Westfield. The children could be more satisfactorily cared for and better discipline could be maintained if they were kept entirely separate from the adult patients, with whom association is not conducive to either mental or moral improvement. That there is need for such a school building, is shown by the fact that there are now in the four sanatoria sixty-nine children from five to sixteen years of age. Applications for the admission of children are rapidly increasing in number, as only recently has their admission been encouraged.

A teacher's task in such a school is many sided, and it will take a woman of more than the usual attainments to be successful as a teacher of books, a player of games and an instructor in the mysteries of nature.

*Our Greatest Asset.* Our national health is physically our greatest national asset. To prevent any possible deterioration of the American stock should be a national ambition. We cannot too strongly insist on the necessity of proper ideals for the family, for simple living and for those habits and tastes which produce vigor and make men capable of strenuous service to their country.—*Theodore Roosevelt.*

*Band Root Abutments.* In regard to bands for the roots, of course such a thing is absolutely essential if you use root abutments for bridges. I regard it as essential in the construction of crowns, for we have observed a number of bad accidents in our own experience from our own cases and in cases of others of fractures of the lateral roots, which have been crowned without using a band. It has come to be our practice to band all lateral roots and band thin bicuspids.—J. E. Nyman in *Review.*

